# Stakeholder consensus for decision making in eye-gaze control technology for children, adolescents and adults with cerebral palsy service provision: findings from a Delphi study

**DOI:** 10.1186/s12883-021-02077-z

**Published:** 2021-02-10

**Authors:** Petra Karlsson, Tom Griffiths, Michael T. Clarke, Elegast Monbaliu, Kate Himmelmann, Saranda Bekteshi, Abigail Allsop, René Pereksles, Claire Galea, Margaret Wallen

**Affiliations:** 1 Cerebral Palsy Alliance Research Institute, Discipline of Child and Adolescent Health, The University of Sydney, Frenchs Forest, PO Box 6427, Sydney, NSW 2086 Australia; 2 Cambridge University Hospital, NHS Foundation Trust, Cambridge, UK; 3 Division of Psychology and Language Sciences, University College London, London, UK; 4 KU Leuven – University of Leuven, Department of Rehabilitation Sciences Campus Bruges, Bruges, Belgium; 5 Department of Pediatrics, Institute of Clinical Sciences, Sahlgrenska Academy, University of Gothenburg, Gothenburg, Sweden; 6 Cerebral Palsy Alliance, Sydney, Australia; 7 School of Allied Health, Australian Catholic University, Sydney, Australia

**Keywords:** Accessibility, Eye-gaze control technology, Cerebral palsy, Disabilities, Clinical decision-making, Assistive technology, Augmentative and alternative communication

## Abstract

**Background:**

Limited research exists to guide clinical decisions about trialling, selecting, implementing and evaluating eye-gaze control technology. This paper reports on the outcomes of a Delphi study that was conducted to build international stakeholder consensus to inform decision making about trialling and implementing eye-gaze control technology with people with cerebral palsy.

**Methods:**

A three-round online Delphi survey was conducted. In Round 1, 126 stakeholders responded to questions identified through an international stakeholder Advisory Panel and systematic reviews. In Round 2, 63 respondents rated the importance of 200 statements generated by in Round 1. In Round 3, 41 respondents rated the importance of the 105 highest ranked statements retained from Round 2.

**Results:**

Stakeholders achieved consensus on 94 of the original 200 statements. These statements related to person factors, support networks, the environment, and technical aspects to consider during assessment, trial, implementation and follow-up. Findings reinforced the importance of an individualised approach and that information gathered from the user, their support network and professionals are central when measuring outcomes. Information required to support an application for funding was obtained.

**Conclusion:**

This Delphi study has identified issues which are unique to eye-gaze control technology and will enhance its implementation with people with cerebral palsy.

**Supplementary Information:**

The online version contains supplementary material available at 10.1186/s12883-021-02077-z.

## Background

Cerebral palsy is an umbrella term that encompasses a group of disorders affecting movement and posture. The primary motor disorder is often accompanied by associated impairments of sensation, cognition, communication, perception, behaviour, and/or by seizure disorder [1]. The type of cerebral palsy and severity of motor involvement play an important role in determining a person’s participation in everyday activities including the ability to speak. For example, population-based studies show that one in two children with cerebral palsy have a speech disorder, and one in three are non-verbal [2]. Speech and motor difficulties may hinder recognition of a child’s cognitive capabilities, potentially leading to inaccurate evaluations of strengths and difficulties [3]. Under-estimating a child’s cognitive ability may limit provision of opportunities for learning and development as family, peers, educators and health professionals may not fully realise or respond appropriately to the child’s developmental profile.

Eye-gaze control technology provides an effective and direct access method to computers and speech generating devices for people with significant physical disabilities, such as cerebral palsy [4, 5], who are able to use purposeful looking behaviours including gaze fixations and gaze transfers [6–10]. This technology holds the potential to unlock people’s capacity to participate in leisure and productivity pursuits, play games, listen to music, use social media, and for environmental control. For individuals with communication difficulties, it can also offer access to specialist software for augmentative and alternative communication (AAC) [8, 10–17]. Eye-gaze control technology involves an infra-red camera, which works in conjunction with specialised software, to monitor and respond to a user’s eye movements [18]. The technology allows a person to move a cursor across as screen and make on-screen selections by deliberately fixing their gaze on a target on a screen.

Although literature is available to guide assistive technology provision [19] and AAC implementation [20, 21], there is almost none available to support decision-making concerning the use of eye-gaze control technology as an access method. Information is required about “who” would be the most appropriate candidates, “what” technology is most effective, “when” it is best introduced, “how” it should be implemented and outcomes measured [22]. This gap in guidance is highlighted by two recent systematic reviews which aimed to identify the effectiveness of eye-gaze control technology as a method of access, assessment and implementation strategies [22] and appropriate outcome measures [7]. Single case studies and small group studies, identified in the reviews, have provided preliminary evidence for use of eye-gaze control technology with children to achieve social and communication goals [6, 11, 23, 24], however, the reviews highlighted a pressing need for research to inform clinical practice and support funding for this technology.

Evidence-based practice draws on the best available evidence, clinical expertise and the values and preferences of recipients of health care [25]. Therefore, this Delphi study aimed to build international consensus from key stakeholders to develop evidence- and consumer-informed clinical guidelines for assessment, trial, implementation, funding and outcome measurement of eye-gaze control technology for children, adolescents and adults with cerebral palsy.

## Methods

### Study design

**Fig. 1:**
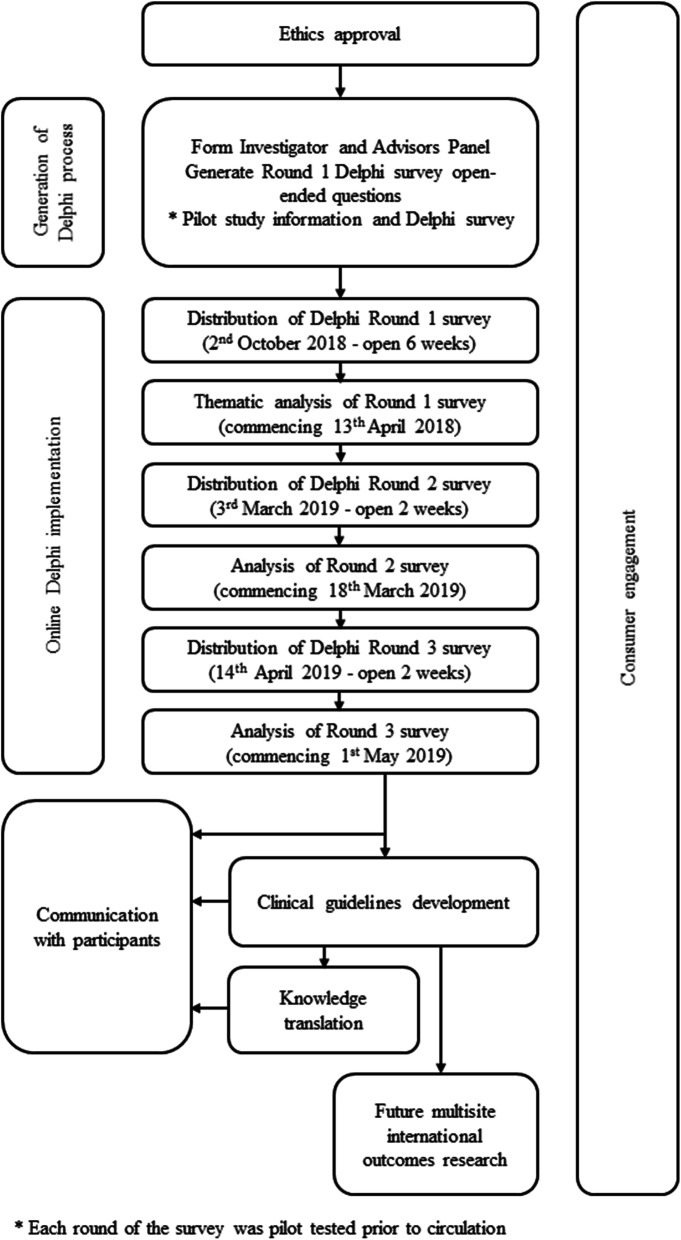
Flowchart of steps involved in completing this project

The Delphi process followed the principles of the Guidance on Conducting and Reporting DElphi Studies (CREDES) [26]. CREDES was developed to provide guidance to researchers conducting Delphi studies on enhancing the rigour and transparency of reporting and to support reproducibility and usefulness of research. CREDES can be used to guide the design of Delphi studies to enhance methodological rigour (Supplementary file 1). A 3-round, online Delphi process was used to identify, and build consensus on, issues that stakeholders viewed as critical to consider when implementing eye-gaze control technology for people with cerebral palsy. Three rounds are conventionally considered sufficient to enable consensus to be reached [26], minimise respondent fatigue [23] and reduce the risk of attrition with further rounds. Three rounds were also feasible within the resources available to the study [24]. In a Delphi study, researchers apply a high degree of methodological rigor to systematically elicit information from respondents [26–29]. Using an iterative process, researchers seek input from stakeholders across a number of rounds of structured questionnaires until group consensus is achieved. The overall study process is presented in Fig. 1 including the dates for each round.

The Delphi process is anonymous and non-hierarchical so it can elicit open and honest views from disparate groups such as clinicians, researchers and, in particular, people with disability and their support network, without group pressure. The process mitigates against individuals feeling deferential towards clinicians and researchers, or inhibited by the presence of more senior individuals or stronger voices [27–31]. As an iterative process it allows minority views to be carefully considered, rather than discarded early in the process [27]. The process also provides opportunities for participants to alter their opinions with the benefit of further consideration, and informed by the collective ideas of the other participants’ anonymous responses [31].

An online Delphi process was a particularly suitable methodology for the current topic as it enabled engagement with large, diverse, international groups of participants. Additionally, it was intended to facilitate inclusion of users of eye-gaze control technology and their communication partners (such as parents, partners, families, caregivers and teachers), as well as clinicians, researchers, industry and funders [26, 28, 30–32].

#### Consumer and stakeholder involvement

In this study “consumers” are defined as people with cerebral palsy who are eye-gaze control technology users and their communication partners, such as family members and caregivers. Consumer involvement was embedded in this study from the start; a mother of an adolescent user of eye-gaze control technology was a study investigator. In addition, an international Advisory Panel, comprised of stakeholders from different fields of interest, informed each stage of the study. See Table 1 for additional details of consumer involvement reported according to the Short Form of Guidance for Reporting Involvement of Patients and the Public 2 (GRIPP2-SF) [33]. These are reporting guidelines developed to enhance the quality, transparency and consistency of consumer involvement in research.

**Table 1:** Consumer involvement – Short Form of the Guidance for Reporting Involvement of Patients and the Public

Section and topic	Description
1: Aim	To incorporate the lived experiences and expertise of consumers into all stages of the study.
2: Methods	The investigator team included a consumer investigator, the mother of an adolescent eye-gaze control technology user. An Advisory Panel was established to collaborate with the investigators to broaden geographical representation and expertise. The Advisory Panel comprised a user of eye-gaze control technology, a parent of a young user of eye-gaze control technology, allied health professionals working as assistive technology consultants specialising in eye-gaze control technology (two occupational therapists, three speech pathologists) and one occupational therapist specialising in functional vision and its assessment. The Advisory Panel and investigator team had members from the United Kingdom, Belgium, Sweden, the United States of America and Australia. The Advisory Panel were purposively invited from amongst the research team’s networks to provide diversity of representation of stakeholder group and geographical location. Consumers were recruited from amongst the researchers’ networks. Consumers signed a Terms of Reference which specified the background to the study, purpose and activities of the Stakeholder Advisory Panel, consumer roles and responsibilities, time frames involved and information about the operation of the Panel. Consumers were involved at each stage of the project including input to the ethics application, reviewing participant information and communications, developing recruitment strategies and circulating the Delphi to their networks, generating items for the Delphi survey, pilot testing and analysis of each round of the Delphi survey, reviewing this publication, and collaborating on knowledge translation. Communication was managed by video/teleconference and email. The consumer investigator co-presented findings at an international conference (funded by research monies) and is a co-author on this paper. The consumer investigator and consumer Advisory Panel members were reimbursed for their time from research funding.
3: Results Outcomes	Consumer involvement had substantial impact on all stages of the research. Comprehension of the recruitment material, participant information and each Delphi survey were improved and additional critical information included to ensure all were informative and accessible. Clear advice was given about reducing the length and improving the format of the surveys to enhance readability and likelihood of participation. Insightful perspectives about interpreting responses to Round 1 resulted in clarity of themes and statements to progress to Round 2. The consumer perspectives on interpreting the findings to progress to the clinical guidelines were highly valuable.
4: Discussion Outcomes	Consumer involvement was central to this study and effectively influenced the quality of each stage. The consumer investigator was involved very early and collaborated in shaping the study and at each stage thereafter.
5: Reflections	This research may have benefited by increased investigator efforts to more closely support consumers to be involved, and to increase the numbers of consumers, particularly users of eye-gaze control technology themselves, to harness greater representation and perspectives, and to have a stronger voice and impact. This support may include research education and taking time to closely communicate with individual consumers to ensure that they could engage as thoroughly as possible. It would also be useful to encourage consumers to communicate amongst themselves to support each other in their roles.

#### Participants and recruitment

Participants were eligible to take part if they were people with cerebral palsy who used eye-gaze control technology, or their family members or caregivers. Other eligible participants were professionals who supported the use of eye-gaze control technology or had been integral in assessing and/or implementing eye-gaze control technology for people with cerebral palsy, specifically, clinicians or technologists, researchers and “others who identified with these inclusion criteria”.

A link to the electronic Delphi survey was embedded in an introductory message circulated amongst the local, national and international electronic networks of the Investigator Team and Advisory Panel, aiming to reach, via a snowball effect, a range of participants across countries and settings. The introduction included a video message from the consumer investigator and her daughter, inviting participation in the study. Participants who consented to participate in the study and met the eligibility criteria were directed to the survey. Respondents who started the survey but indicated they met the “other” category and not a category for inclusion (i.e., a person with cerebral palsy, a family member or caregiver or a professional supporting eye-gaze control technology for people with cerebral palsy) were thanked for their interest and exited from the survey and study. In subsequent rounds, surveys were sent only to people who had completed the previous round.

Participant demographics were gathered in Round 1 of the survey. An email address was obtained from each participant at each round of the survey to facilitate reminders and distribution of subsequent rounds of the survey. The email address was used to link with participant demographics collected in Round 1 to enable description of the unique sub-sample responding to subsequent rounds.

### Procedures

#### Generation of content for round 1

As we had identified that there was little evidence to guide practice, open-ended questions were used in Round 1 to identify statements which respondents believed should be included in clinical guidelines. The questions were developed with the aim of identifying issues related to all aspects of eye-gaze control technology, spanning identification of the unique contribution and challenges eye-gaze control technology offers as an access method, assessing readiness for eye-gaze control technology, assessment and outcome measurement, effective strategies for implementing eye-gaze control technology, communication partner instruction, costs and funding [16]. Over a series of meetings, the Investigator Team and Advisory Panel identified 17 open-ended questions, organised in seven topic areas (Supplementary file 2) [26]. The questions were written in English and translated and back-translated to enable distribution in English, Swedish, Dutch and French – the languages spoken by the Investigator Team and Advisory Panel.

#### Survey platform and pilot testing

Research Electronic Data Capture (REDCap - hosted at University of Sydney) was used as the online platform to collect responses. This system provides a secure, web-based application designed to support data capture for research studies.

Pilot testing of each round of a Delphi survey is considered best practice [26, 28, 29]. The draft survey of each round (including study information, items collecting participant characteristics, questions/statements) was therefore developed, tested in its online form and modified iteratively by the Investigator Team and Advisory Panel until agreement was reached that the survey was rigorous, unambiguous and able to elicit meaningful responses. Usability on a variety of web browsers on Mac and Windows-based computers, as well as Android and iOS-based mobile devices [32] was also checked prior to dissemination of the survey link. The survey was prepared in accordance with accessibility standards for people with visual impairment and limited access.

#### Responses to round 1 of the Delphi survey

The narrative responses to Round 1 were thematically analysed and the resultant statements, categorised by theme, were disseminated in Round 2.

#### Rating of the importance of the statements in rounds 2 and 3 of the Delphi survey

Respondents were asked to rate all statements resulting from Round 1 in response to the question: *How important is this item to include in clinical guidelines*? Statements were rated on a 9-point scale where 1–3 corresponded to low importance, 4–6 represented statements that were considered important but not critical, and statements rated 7–9 were considered to be of critical importance. An alternative response option (labelled: don’t know or prefer not to answer) was also provided. Participants were offered free-text options to expand or comment on any aspect of their responses. A personalised reminder email was sent to non-responders 1 week prior to closing Round 2 to optimise survey return rate. In this round, surveys were offered in English and Swedish, based on the language of the majority of respondents from Round 1.

Any additional statements identified in the free text boxes during Round 2, and which did not duplicate existing content, were analysed thematically and added as new statements under the appropriate theme. Statements rated 7–9 by ≥70% of respondents and 1–3 by < 15% of respondents were considered as reaching consensus, other statements were discarded [34]. The process of identifying consensus statements was completed separately for two groups of participants, all participants together and people with cerebral palsy and their families and caregivers. All statements reaching consensus from both these groups progressed to Round 3. This process ensured that the most important content from people with cerebral palsy and their families and caregivers was included for ongoing consideration. In the third round, surveys were offered in English and Swedish.

### Data analysis

The thematic qualitative analysis of narrative responses to Round 1 was performed independently by three investigators (PK, TG, MW) [35]. The investigators triangulated their responses to reach consensus on themes, theme titles and statements contained within each theme to enhance trustworthiness of analyses [31]. To further minimise bias, the thematic analysis was reviewed by the Investigator Team and Advisory Panel and revised in an iterative manner until panel members confirmed consensus [27]. This process was in lieu of member checking with the respondents which we avoided in order to minimise burden, given the already substantial request to complete 3-rounds of Delphi surveys. The resultant statements, categorised by theme, were disseminated in Round 2.

SPSS V.25 [36] was used to generate descriptive statistics of participant characteristics and calculate level of consensus for each statement rated by participants in Rounds 2 and 3.

## Results

### Round 1

One hundred and twenty-six participants completed Round 1 of the Delphi survey (Tables 2 and 3). Twenty additional people were exited from the survey after indicating that they did not meet the inclusion criteria for an eligible participant. The total number of potential participants who received invitations to participate is unknown due to the snowball nature of recruitment and, therefore, a response rate could not be calculated for Round 1.

**Table 2:** Gender, language used by participants, residential area, profession and highest level of education

	Round 1 *N* = 126% (n)	Round 2 *N* = 62% (n)	Round 3 *n* = 41% (n)
Gender (female)	86.5 (109)	86.9 (54)	87.8 (36)
Language			
English	86.5 (109)	87.1 (54)	87.8 (36)
Swedish	11.1 (14)	1.6 (1)	12.2 (5)
French	1.6 (2)	Not offered in French	Not offered in French
Dutch	0.8 (1)	Not offered in Dutch	Not offered in Dutch
Residential area			
City	52 (65)	61.3 (38)	70.7 (29)
Regional	28.8 (36)	22.6 (14)	14.6 (6)
Metropolitan	17.6 (22)	14.5 (9)	12.2 (5)
Remote	1.6 (2)	1.6 (1)	2.4 (1)
Countries	Great Britain (38%), Australia (15%), Sweden (15%), USA (14%), Belgium (5%), Canada (3%) and one participant each from Afghanistan, Croatia, India, Latvia, Malaysia, Norway, Slovenia, Spain, Turkey, Virgin Islands and Zimbabwe.	Great Britain (31%), Sweden (18%), Australia (16%), USA (13%), Belgium (5%), Canada (5%) and 1 participant each from Afghanistan, Croatia, India, Malaysia, Norway, Slovenia, and Ireland.	Great Britain (29%), Sweden (20%), Australia (15%), USA (10%), Belgium (5%), Canada (3%) and one participant each from Afghanistan, Croatia, India, Malaysia, Norway, Slovenia, and Ireland.
Profession			
Clinicians/technologists	71.2 (89) ^a^	72.6 (45)	73.2 (30)
*Disciplines*			
Occupational therapist	24.7 (22)	20.4 (10)	20 (6)
Speech pathologist	59.6 (53)	55.1 (27)	53 (16)
Biomedical engineer	2.2 (2)	2.0 (1)	3.3 (1)
Medical practitioner	4.5 (4) ^b^	4.1 (2)	6.7 (2)
Other	9.0 (8) ^c^	10.2 (5)	16.7 (5)
Educators	11.2 ^d^ (14)	9.7 (6)	4.9 (2)
Researcher	2.4 (3)	4.8 (3)	7.3 (3)
*Disciplines*			
Occupational therapist	*n* = 2	*n* = 2	*n* = 2
Optometrist	*n* = 1	*n* = 1	*n* = 1
Other involved with eye-gaze control technology	3.2 (4)	3.2 (2)	4.9 (2)
*Disciplines*			
Medical practitioner	*n* = 2	*n* = 2	*n* = 2
Speech pathologist	*n* = 1	*n* = 0	*n* = 0
Unspecified	*n* = 1	*n* = 0	*n* = 0
Highest level of education			
Tertiary	80.2 (101)	79.0 (49)	78.0 (32)
Certificate qualifications	17.5 (22)	17.7 (11)	19.5 (8)
Secondary	2.4 (3)	3.2 (2)	2.4 (1)
Age groups the participants supported with eye-gaze control technology ^e^			
< 6 years	23 (87)	21 (47)	23 (31)
7–14 years	26 (101)	23 (53)	26 (36)
15–24 years	24 (92)	22 (50)	24 (33)
25–44 years	11 (42)	13 (30)	11 (15)
45–64 years	9 (34)	11 (26)	9 (12)
> 65 years	7 (29)	10 (22)	7 (10)

^a^*n* = 1 did not respond

^b^ Fields of practice for medical practitioners were: Round 1 - paediatrician (neurodisability, neurodevelopment; *n* = 3), child neurologist (*n* = 2) and physical medicine and rehabilitation (*n* = 1); Round 2 - paediatrician (neurodisability; *n* = 2), child neurologist (*n* = 1); and Round 3 - paediatrician (neurodisability; *n* = 2), child neurologist (*n* = 1). Note: some participants nominated two fields of specialty

^c^ The “other” clinicians – were: Round 1 - assistive technology specialist (*n* = 5), electronic engineer (*n* = 1), orthoptist (*n* = 1), psychologist (*n* = 1) and therapy assistant (*n* = 1) [Note: One participant specified more than area]; Round 2 - assistive technology specialist (*n* = 2), electronic engineer (*n* = 1), orthoptist (*n* = 1), psychologist (*n* = 1) and AAC specialist (*n* = 1); and Round 3 - assistive technology specialist (*n* = 2), electronic engineer (*n* = 1), psychologist (*n* = 1) and AAC specialist (*n* = 2)

^d^ The work environments of educators were: Round 1 - special schools (*n* = 3), itinerant roles supporting students in various schools (*n* = 2) and mainstream school (*n* = 1); Round 2 – mainstream school (*n* = 1), special school (*n* = 2) and Round 3 - special schools (*n* = 2)

^e^ Some participants nominated two or more age groups

**Table 3:** Characteristics of participants

	Round 1 *N* = 126% (n)	Round 2 *N* = 62% (n)	Round 3 *n* = 41% (n)
Respondents			
Family/caregivers	9.6 (12)	8.1 (5)	9.8 (4)
Person with cerebral palsy	2.4 (3)	1.6 (1)	0
Classification of functioning ^a^			
GMFCS E&R			
Level V	*n* = 3	*n* = 1	n/a ^c^
MACS			
Level V	*n* = 2	*n* = 0	n/a
Level IV	*n* = 1	*n* = 1	n/a
CFCS			
Level IV	*n* = 2	*n* = 1	
Level II	*n* = 1	*n* = 0	n/a
VSS			
Level III	*n* = 2	*n* = 1	n/a
Level I	*n* = 1	*n* = 0	n/a
	Round 1 Mean Range SD	Round 2 Mean Range SD	Round 3 Mean Range SD
Age			
Family/caregivers	48.1	47.4	46.5
	36–62	37–54	36–54
	7.7	6.3	7.6
Person with cerebral palsy	58.0	20 ^b^	n/a ^c^
	54–61		
	3.6		
Clinicians/technologists	41.4	42.2	43.2
	22–67	22–67	22–66
	11.4	11.6	11.9
Educators	40.8	38	37
	22–63	22–55	25–49
	16.1	15.8	17.0
Researchers	43.7	43.7	43.7
	29–52	29–52	29–52
	12.7	12.7	12.7
Other involved with eye-gaze control technology	48.9	66	66
	22–75	57–75	57–75
	16.8	12.7	12.7
Experience of using/ communication with eye-gaze control technology (Years)			
Family/caregivers	3.5	3.5	2.75
	< 1 to 6	1 to 6	< 1 to 6
	2.1	2.2	2.8
Person with cerebral palsy	2.3	9 ^b^	n/a ^c^
	0–5 years		
	2.5		

^a^ *GMFCS E&R* Gross Motor Function Classification System Expanded and Revised [37]; *MACS* Manual Ability Classification System [38]; *CFCS* Communication Function Classification System [39]; *VSS* Viking Speech Scale [40]

^b^ Only one person with cerebral palsy in this round

^c^ No people with cerebral palsy in this round

From the 17 questions 1995 codable units were derived. These were discrete responses to survey questions which were then collated into 200 thematically uniform statements. These 200 statements were clustered under eight themes; including: (1) Unique features of eye-gaze control technology as an access method; (2) Initial assessment prior to a trial; (3) Decision making when conducting a trial of eye-gaze control technology; (4) Learning, practise and support requirements when implementing this technology; (5) Evaluation of outcomes and measures to use; (6) Frequency of follow-up and review; (7) Informing funding applications and bodies, and (8) Preparation for possible future access to eye-gaze control technology if it is not currently appropriate. Several issues were common to these eight themes. Table 4 contains these themes, the corresponding statements and the ratings given to each statement in subsequent rounds.

**Table 4:** Statements and the ratings given to each statement in subsequent rounds

Statements identified in Round 1^**a**^		Round 2 ^**b**^ % of respondents			Round 3 ^**b**^ % of respondents		
		Critical importance	Important	Not critical	Critical importance	Important	Not critical
**Question 1: What is unique about eye-gaze control technology as an access method?**							
For the person	**[1.3.1.a] it can be suitable if they have severe motor disabilities which limits voluntary gross and fine motor control**	**87.9**	**10.3**	**1.7**	**92.7**	**7.3**	**0.0**
	**[1.2.1.b] it can be a fast, efficient and accurate direct access method to the computer as well as to communication software**	**67.2** ^**d**^	**29.3**	**3.4**	**82.9**	**17.1**	**0.0**
	it can provide the opportunity to communicate spontaneous, novel words quickly and efficiently	69.0	24.1	6.9			
	it can be fatiguing	44.8	37.9	17.2			
	**[1.3.1.c] it can be reliant on seating and positioning**	**74.1**	**15.5**	**10.3**	**82.9**	**17.1**	**0.0**
	**[1.3.1.d] it may require careful set up and positioning prior to use**	**79.3**	**17.2**	**3.4**	**85.4**	**14.6**	**0.0**
	**[1.3.1.e] it may be difficult to use outdoors due to bright sunlight**	**57.4** ^**d**^	**35.2**	**7.4**	**79.5**	**17.9**	**2.6**
For the person’s support network	it can be hard to demonstrate/model how to use the device in a clear way	43.1 ^**d**^	44.8	12.1	63.4	31.7	4.9
**Question 2: What information should be considered as part of an eye-gaze control technology assessment** **before a trial?**							
Ability to visually attend to the screen	**[2.3.1.a] vision (acuity)**	**74.1**	**20.7**	**5.2**	**78.0**	**19.5**	**2.4**
	**[2.3.1.b] visual motor (eye movements)**	**86.0**	**10.5**	**3.5**	**87.8**	**9.8**	**2.4**
	**[2.3.1.c] visual perceptual skills**	**76.8**	**16.1**	**7.1**	**77.5**	**17.5**	**5.0**
Ability to show understanding	cognition	41.1	42.9	16.1			
	understanding of cause and effect	46.6	32.8	20.7			
	attention	50.0	43.1	6.9			
	concentration	44.8	48.3	6.9			
	memory	24.1	62.1	13.8			
	communicative intent	49.1	42.1	8.8			
	existing communication methods	43.6	38.2	18.2			
	understanding of language	39.3	42.9	17.9			
General health issues	physical health	24.6	59.6	15.8			
	medications	33.9	41.1	25.0			
	fatigue	50.0	41.4	8.6			
	pain	36.2	51.7	12.1			
	hearing	29.3	39.7	31.0			
Motor ability	head control	55.2 ^**d**^	37.9	6.9	58.5	34.1	7.3
	**[2.3.2.a] positioning**	**84.5**	**15.5**	**0.0**	**80.5**	**17.1**	**2.4**
Drive, ambitions and experiences	motivation	62.1	31.0	6.9			
	personal preferences	53.6	35.7	10.7			
	personal goals	47.4	36.8	15.8			
	previous trials or use of technology and the outcomes	28.1	49.1	22.8			
	interest in technology	24.1	41.4	34.5			
**Question 2a: The following information about the person’s** **environment and support network** **should be considered as part of an eye-gaze control technology assessment before a trial.**							
In the environment	lighting levels in settings where the device will be used	50.9	45.6	3.5			
	what other technology the device might need to interface with	52.6	45.6	1.8			
	**[2.3.3.a] the need to mount the device, and if so, how**	**86.2**	**13.8**	**0.0**	**85.4**	**14.6**	**0.0**
	**[2.3.3.b] whether the technology will need to be moved between settings**	**74.1**	**22.4**	**3.4**	**77.5**	**20.0**	**2.5**
The support network	their expectations	58.6	36.2	5.2			
	**[2.3.4.a] their need for support and training**	**89.5**	**10.5**	**0.0**	**90.2**	**9.8**	**0.0**
	**[2.3.4.b] who the key people are that will coordinate the implementation the upcoming trial**	**90.9**	**9.1**	**0.0**	**92.7**	**7.3**	**0.0**
	**[2.3.4.c] their ability to borrow or rent one or more devices to trial for a sufficient period of time**	**72.7**	**16.4**	**10.9**	**70.0**	**22.5**	**7.5**
	**[2.3.4.d] their availability to support access, use and/or teach the person to use their device**	**87.7**	**12.3**	**0.0**	**87.8**	**12.2**	**0.0**
	their knowledge of the application process for any available funding	45.5	41.8	12.7			
**Question 2b: The following information about the** **technical aspects** **of eye gaze-control technology should be considered as part of an assessment before a trial.**							
The eye-gaze control technology system	**[2.3.5.a] ability to be used with different light levels or outside**	**45.6** ^**d**^	**45.6**	**8.8**	**57.5** ^**d**^	**37.5**	**5.0**
	battery life of computer/speech generating device interfacing with the eye-gaze control technology camera	64.3 ^**d**^	32.1	3.6	62.5	37.5	0.0
	memory of computer/speech generating device interfacing with the eye-gaze control technology camera	50.9	41.5	7.5			
	**[2.3.5.b] portability**	**65.5** ^**d**^	**31.0**	**3.4**	**73.2**	**26.8**	**0.0**
	**[2.3.5.c] ease of set up**	**70.7**	**27.6**	**1.7**	**78.0**	**22.0**	**0.0**
	options for methods of calibration	67.3 ^**d**^	30.9	1.8	63.4	31.7	4.9
	compatibility with other hardware and software	63.2	31.6	5.3			
	**[2.3.5.d] connectivity with the computer/speech generating device interfacing with the eye-gaze control technology camera using WiFi, Bluetooth and InfraRed**	**65.4** ^**d**^	**30.8**	**3.8**	**71.8**	**25.6**	**2.6**
	screen size of computer/speech generating device interfacing with the eye-gaze control technology camera	57.9	42.1	0.0			
	volume of computer/speech generating device interfacing with the eye-gaze control technology camera	53.6	42.9	3.6			
	weight of computer/speech generating device interfacing with the eye-gaze control technology camera	49.1	45.6	5.3			
The device and its flexibility to respond to the person’s ability	**[2.3.6.a] hold deliberate fixations (dwell time)**	**82.1**	**16.1**	**1.8**	**85.4**	**12.2**	**2.4**
	**[2.3.6.b] calibrate**	**77.2**	**19.3**	**3.5**	**80.5**	**17.1**	**2.4**
	activate sleep mode and move between programs, once the device is set up	49.1	41.5	9.4			
	**[2.3.6.c] use vision despite limitations (vision in only one eye, vision out of the corner of the eye, shifting dominance of the eyes)**	**83.3**	**16.7**	**0.0**	**100**	**0.0**	**0.0**
**Question 3: The following factors should inform decision making when carrying out a trial**							
The person’s	health and well-being to enable consistent participation in trial	54.4	38.6	7.0			
	motivation	68.4	26.3	5.3			
	**[3.3.1.a] preferences**	**71.7**	**26.4**	**1.9**	**72.5**	**27.5**	**0.0**
	**[3.3.1.b] goals for the trial, jointly established with the person, their family and clinician**	**82.5**	**17.5**	**0.0**	**87.5**	**12.5**	**0.0**
	**[3.3.1.c] seating and positioning equipment are adequate to meet their needs**	**93.0**	**7.0**	**0.0**	**90.2**	**9.8**	**0.0**
The person’s support network, specifically their	ability to be flexible in responding when a person’s skills advance or their abilities decline	63.2	33.3	3.5			
	strategies to minimise the person’s fatigue	64.3	35.7	0.0			
	**[3.3.2.a]. attitudes**	**82.5**	**12.3**	**5.3**	**85.4**	**12.2**	**2.4**
	**[3.3.2.b] training needs (the person’s support network needs to be comfortable in implementing the trial, operating the device, using and modifying software, and using communication partner strategies)**	**98.2**	**1.8**	**0.0**	**87.8**	**9.8**	**2.4**
	**[3.3.2.c] input and feedback (family, carers, teachers, employers and others in the settings in which the device is trialled)**	**91.2**	**7.0**	**1.8**	**90.0**	**7.5**	**2.5**
	**[3.3.2.d] ability to provide regular and sufficient time to practise**	**89.5**	**10.5**	**0.0**	**95.1**	**4.9**	**0.0**
	**[3.3.2.e] access to local therapists to support the device trial**	**84.2**	**14.0**	**1.8**	**80.5**	**19.5**	**0.0**
	**[3.3.2.f] access to staff who are experienced with using and teaching eye-gaze control technology and are motivated and engaged in an ongoing way to fully support the person and their support network with the device trials**	**78.9**	**17.5**	**3.5**	**85.4**	**14.6**	**0.0**
The person’s environment	settings in which a person will use a device	66.1	32.1	1.8			
	**[3.3.3.a] appropriate space which is large enough to accommodate the device and mountings, and which will support use of device (e.g., quiet)**	**70.4**	**27.8**	**1.9**	**73.2**	**24.4**	**2.4**
	**[3.3.3.b] mounting options (e.g., wheelchair, table or floor mounting)**	**76.8**	**23.2**	**0.0**	**75.6**	**22.0**	**2.4**
	**portability of the device**	**68.5** ^**d**^	**24.1**	**7.4**	**73.2**	**26.8**	**0.0**
	**[3.3.3.c] access to low-tech AAC for periods when using eye-gaze devices is not possible or practical**	**78.0**	**18.0**	**4.0**	**78.0**	**19.5**	**2.4**
	**[3.3.3.d] customisation of the device, for example, the on-screen cell size**	**88.9**	**11.1**	**0.0**	**90.0**	**10.0**	**0.0**
	**[3.3.3.e] reliability of the eye-gaze control system**	**96.4**	**3.6**	**0.0**	**100.0**	**0.0**	**0.0**
	**[3.3.3.f] features of the system, such as screen size, on-switch location and accessibility, battery life, size and weight, ease of installation and set up**	**75.0**	**25.0**	**0.0**	**85.4**	**12.2**	**2.4**
	**[3.3.3.g] fun and motivating activities which are intuitive to use; appropriate to the goals, abilities and interests of the person; and have varying levels to enable progression**	**85.7**	**14.3**	**0.0**	**87.8**	**12.2**	**0.0**
	**[3.3.3.h] sufficient time is necessary to properly consider all the variables that might impact on the outcome of the trial**	**80.4**	**19.6**	**0.0**	**92.7**	**7.3**	**0.0**
	**[3.3.3.i] if trialling more than one system, uniform trial conditions are required to enable equal comparisons**	**65.5** ^**d**^	**34.5**	**0.0**	**73.2**	**26.8**	**0.0**
	**[3.3.3.j] funding to hire or rent devices, fund staff for trials and purchase a device if appropriate**	**66.7** ^**d**^	**24.1**	**9.3**	**75.7**	**16.2**	**8.1**
	**[3.3.3.k] keeping up to date with the latest information on eye-gaze research and technology developments**	**75.0**	**23.2**	**1.8**	**78.0**	**19.5**	**2.4**
The procedures in place	ongoing monitoring to record progress and outcomes	77.2	19.3	3.5	68.3	31.7	0.0
	**[3.3.4.a] building on user’s abilities, skills, development and progress**	**82.5**	**17.5**	**0.0**	**87.8**	**12.2**	**0.0**
	**[3.3.4.b] a plan for the device trial, with clearly outlined roles and responsibilities**	**80.4**	**19.6**	**0.0**	**82.9**	**17.1**	**0.0**
**Question 4a: When selecting** **activities and resources** **that are useful for the person and their support network to learn to use the eye-gaze control technology effectively, the following should be considered.**							
Games and activities	commercially available in skill building software and which drives practise and monitors progress	58.5	35.8	5.7			
	a range of engaging activities controlling their surroundings such as music and TV	53.7	38.9	7.4			
	low-tech form (e.g., E-Tran frame)	50.0	37.0	13.0			
Educational activities to achieve	literacy	52.8 ^**d**^	35.8	11.3	50	37.5	12.5
	vocabulary	71.7	24.5	3.8	65.0	30.0	5.0
	general learning activities	60.4	35.8	3.8			
Resources	online resources, for example, training videos, blogs and e-learning	53.7	38.9	7.4			
	**[4.3.1.a] guidelines for the assessment of eye pointing**	**68.5** ^**d**^	**25.9**	**5.6**	**74.4**	**23.1**	**2.6**
**Question 4b: Information to consider when learning how to use the eye-gaze control technology -** **Practising using the technology** **to learn to use it effectively**							
The person’s practise should take place	**[4.3.2.a] in regular, frequent sessions, please specify:**	**82.0**	**16.0**	**2.0**	**92.7**	**7.3**	**0.0**
	daily	64.2	34.0	1.9			
	**[4.3.2.b] in short sessions, to reduce the effects of fatigue, please specify:**	**72.5**	**23.5**	**3.9**	**85.4**	**14.6**	**0.0**
in addition, the person’s support network (communication partners)	**[4.3.3.a] should receive regular training and support**	**79.2**	**18.9**	**1.9**	**92.7**	**7.3**	**0.0**
**Question 4c: When recommending practise and support when learning how to use the eye-gaze control technology, the following should be considered**							
The person should	**[4.3.4.a] start by focusing on skills (e.g., cause and effect, targeting, dwell) through activities or games**	**57.7** ^**d**^	**38.5**	**3.8**	**82.9**	**14.6**	**2.4**
	be provided training in technical aspects of the device	36.0	44.0	20.0			
	**[4.3.4.b] be supported by professionals who will guide direction and identify areas to work on**	**88.5**	**11.5**	**0.0**	**95.1**	**4.9**	**0.0**
	**[4.3.4.c] receive face-to-face support in the their usual settings**	**80.4**	**17.6**	**2.0**	**85.4**	**14.6**	**0.0**
	**[4.3.4.d] be provided with clear modelling/demonstration and direction**	**88.2**	**11.8**	**0.0**	**97.5**	**2.5**	**0.0**
	**[4.3.4.e] have an individualised practise plan**	**71.2**	**25.0**	**3.8**	**80.5**	**19.5**	**0.0**
	**[4.3.4.f] have practise embedded in their current routines**	**84.6**	**15.4**	**0.0**	**95.1**	**4.9**	**0.0**
The support network to should receive	**[4.3.5.a] training in technical aspects of the device**	**76.9**	**21.2**	**1.9**	**85.4**	**12.2**	**2.4**
	**[4.3.5.b] timely technical support in person / by phone / online**	**88.5**	**11.5**	**0.0**	**92.7**	**7.3**	**0.0**
	**[4.3.5.c] support from multiple professionals (Speech Language Therapist/Speech pathologist, Occupational Therapist, Physiotherapist/ Physical Therapist, Psychologist, Assistive Technology consultant, Education Professional)**	**80.8**	**19.2**	**0.0**	**87.8**	**12.2**	**0.0**
	face-to-face support in their usual settings	63.5	32.7	3.8			
**Question 5: When and how often should use of eye-gaze control technology be reviewed?**							
During the initial stages - while learning to use the system, review should take place	daily	18.4	32.7	49.0			
	weekly	57.1	32.7	10.2			
	monthly	68.1	23.4	8.5			
	every 3 months/once a school semester (term)	50.0	25.0	25.0			
	6-monthly	43.8	29.2	27.1			
	annually	53.2	21.3	25.5			
	**[5.3.1.a] as required or requested**	**83.7**	**10.2**	**6.1**	**90.2**	**9.8**	**0.0**
During the intermediate stages - while mastering the system, review should take place	daily	12.5	29.2	58.3			
	weekly	24.0	38.0	38.0			
	monthly	43.8	31.3	25.0			
	every 3 months/once a school semester (term)	61.7	19.1	19.1			
	6-monthly	52.2	19.6	28.3			
	annually	53.2	19.1	27.7			
	**[5.3.2.a] as required or requested**	**85.7**	**6.1**	**8.2**	**90.2**	**9.8**	**0.0**
During the latter stages - once use of the system is mastered, review should take place	daily	4.3	10.6	85.1			
	weekly	6.4	10.6	83.0			
	monthly	20.8	22.9	56.3			
	every 3 months/once a school semester (term)	28.6	32.7	38.8			
	6-monthly	56.3 ^**d**^	25.0	18.8	56.1	34.1	9.8
	annually	68.8 ^**d**^	22.9	8.3	64.1	28.2	7.7
	**[5.3.3.a] as required or requested**	**91.8**	**4.1**	**4.1**	**87.8**	**9.8**	**2.4**
**Question 6: Recommendations for measuring the outcomes of eye-gaze control technology**							
Goal attainment	**[6.3.1.a] reports from the person and their support network**	**91.8**	**6.1**	**2.0**	**87.8**	**12.2**	**0.0**
	**[6.3.1.b] observation by professionals**	**83.7**	**14.3**	**2.0**	**87.8**	**9.8**	**2.4**
	Canadian Occupational Performance Measure (COPM)	34.5	34.5	31.0			
	Goal Attainment Scaling (GAS)	56.8 ^**d**^	29.7	13.5	58.3	41.7	0.0
Satisfaction	**[6.3.2.a] reports from the person and their support network**	**91.8**	**8.2**	**0.0**	**97.6**	**2.4**	**0.0**
	**[6.3.2.b] observation by professional**	**75.5**	**20.4**	**4.1**	**78.0**	**19.5**	**2.4**
	Quebec User Evaluation of Satisfaction with assistive Technology (QUEST)	26.1	47.8	26.1			
Functional independence, well-being and quality of life	**[6.3.3.a] reports from the person and their support network**	**85.7**	**14.3**	**0.0**	**97.6**	**2.4**	**0.0**
	**[6.3.3.b] observation by professional**	**75.5**	**22.4**	**2.0**	**87.8**	**12.2**	**0.0**
	the Family Impact of Assistive Technology Scale for Augmentative and Alternative Communication (FIAT-AAC)	37.5 ^**d**^	33.3	29.2	59.3	40.7	0.0
	the Therapy Outcome Measure – AAC (TOM-AAC)	41.4	37.9	20.7	59.3	40.7	0.0
Communication	**[6.3.4.a] reports from the person and their support network**	**93.9**	**6.1**	**0.0**	**95.1**	**4.9**	**0.0**
	**[6.3.4.b] observation by professional**	**85.7**	**14.3**	**0.0**	**87.8**	**12.2**	**0.0**
	C.O.D.E.S Framework	34.8	43.5	21.7			
	Communication Matrix	35.7	42.9	21.4			
	Dynamic AAC Goals Grid-2 (DAGG-2)	33.3	45.8	20.8			
	standardised and norm-based language and communication assessments carried out by a speech and language therapist to monitor progress over time	39.0	43.9	17.1			
	the Pragmatics Profile for People who use AAC	22.6	54.8	22.6			
	the AAC Profile™ – A Continuum of Learning	32.1	50.0	17.9			
Eye-gaze skills	**[6.3.5.a] reports from the person and their support network**	**81.6**	**18.4**	**0.0**	**95.1**	**4.9**	**0.0**
	**[6.3.5.b] observation by professional**	**83.7**	**16.3**	**0.0**	**95.1**	**4.9**	**0.0**
	measures of speed	27.7	61.7	10.6			
	recordings of concentration times	23.4	59.6	23.4			
	Inclusive EyeGaze Learning Curves	12.5	43.8	43.8			
	in-house produced check-list of operational skills	30.0	52.5	17.5			
	pre and post games to evaluate progress	33.3	44.4	22.2			
	Tobii Dynavox Eye Gaze Pathways	18.8	37.5	43.8			
Determining classifications	Communication Function Classification Scale	43.8	37.5	18.8			
	Functional Communication Classification System	37.0	40.7	22.2			
	Eye Pointing Classification Scale	41.4	41.4	17.2			
**Question 7: Funding an eye-gaze control technology system - the following criteria could be considered to inform a supplier, purchaser or funding body of the need for eye-gaze control technology**							
The procedures in place	**[7.3.1.a] recommendations from professionals based on evidence collected during a trial of devices**	**89.4**	**10.6**	**0.0**	**92.7**	**4.9**	**2.4**
	**[7.3.1.b] the application meets the criteria of the supplier, purchaser or funding body**	**82.2**	**15.6**	**2.2**	**87.2**	**12.8**	**0.0**
	the preferred device offers value for money when compared with other options	60.9	28.3	10.9			
	trials of devices were carried out over a reasonable period of time (e.g., 1 to 3 months)	61.7	27.7	10.6			
Outcomes for the person	**[7.3.2.a] enables them to engage in play, leisure and recreation activities**	**83.0**	**17.0**	**0.0**	**82.9**	**17.1**	**0.0**
	**[7.3.2.b] enables them to participate in learning, study and education**	**85.1**	**14.9**	**0.0**	**92.7**	**7.3**	**0.0**
	**[7.3.2.c] enables them to participate in employment and volunteer roles**	**78.3**	**19.6**	**2.2**	**82.9**	**14.6**	**2.4**
	**[7.3.2.d] enables access to a computer and other technology**	**73.3**	**24.4**	**2.2**	**78.0**	**22.0**	**0.0**
	**[7.3.2.e] enables them to control their environment**	**71.7**	**23.9**	**4.3**	**78.0**	**22.0**	**0.0**
	enables them to move around in the environment	47.8	43.5	8.7			
	**[7.3.2.f] increases their ability to communicate**	**93.6**	**6.4**	**0.0**	**97.6**	**2.4**	**0.0**
	**[7.3.2.g] enhances their quality of life and well-being**	**93.6**	**6.4**	**0.0**	**95.1**	**4.9**	**0.0**
	**[7.3.2.h] facilitates their social interaction**	**93.6**	**6.4**	**0.0**	**97.6**	**2.4**	**0.0**
	is the only form of access method is suitable	40.0	33.3	26.7			
	enhances their posture	23.3	53.5	23.3			
	enhances their personal comfort	40.5	42.9	16.7			
	was trialled, and can be used, in a variety of settings	65.2	32.6	2.2			
	demonstrates that they are motivated to use the device	67.4	23.9	8.7			
	**[7.3.2.i] demonstrates that they are satisfied with the device**	**69.6**	**19.6**	**10.9**	**73.2**	**24.4**	**2.4**
	**[7.3.2.j] demonstrates that their goals were achieved**	**69.6**	**21.7**	**8.7**	**73.2**	**24.4**	**2.4**
	**[7.3.2.k] demonstrates necessary skills and abilities to operate eye-gaze control technology, for example, eye-pointing, dwell on a target on screen, language and cognition**	**71.1**	**20.0**	**8.9**	**70.7**	**26.8**	**2.4**
	demonstrated that they will benefit from eye-gaze control technology for a significant period of time	59.6	25.5	14.9			
The person’s support network capacity	**[7.3.3.a] availability of professional staff to provide support**	**83.0**	**12.8**	**4.3**	**90.2**	**9.8**	**0.0**
	**[7.3.3.b] availability to provide support at home, school and other settings**	**76.6**	**19.1**	**4.3**	**90.2**	**9.8**	**0.0**
**Question 8: Preparing for eye-gaze control technology when it is not currently the right choice**							
For the person	**[8.3.1.a] ensure optimal seating, positioning and head support**	**91.7**	**8.3**	**0.0**	**92.5**	**7.5**	**0.0**
	arrange for an eye control assessment	59.6	36.2	4.3			
	arrange for a vision assessment	64.6	25.0	10.4			
	arrange for a cognitive or developmental abilities assessment	40.4	42.6	17.0			
	support skill development such as visual attention, eye pointing, gaze shifting and gaze tracking	65.2	32.6	2.2			
	support the person’s understanding of cause and effect	62.5	35.4	2.1			
	**[8.3.1.b] build the person’s confidence in communicating**	**79.2**	**20.8**	**0.0**	**87.5**	**12.5**	**0.0**
	**[8.3.1.c] encourage the person to use their eyes for making choices**	**81.3**	**18.8**	**0.0**	**82.5**	**17.5**	**0.0**
The person’s support network capacity	**[8.3.1.d] provide practise using low tech options, accessed by eye gaze, to develop communication and language**	**77.1**	**18.8**	**4.2**	**77.5**	**20.0**	**2.5**
	provide practise with lower cost/free software and hardware	36.2	42.6	21.3			
	provide practise with commercially available software to train and develop eye-gaze skills	39.6	43.8	16.7			
	encourage the person in turn-taking	45.7	47.8	6.5			
	**[8.3.1.e] establish clear goals**	**75.0**	**25.0**	**0.0**	**77.5**	**22.5**	**0.0**
	**[8.3.2.a] ensure that all stakeholders keep working together**	**85.4**	**14.6**	**0.0**	**92.5**	**7.5**	**0.0**
	support engagement with other users and support networks	55.3	40.4	4.3			
	**[8.3.2.b] support communication partners to assist people in building the skills to use eye-gaze control technology**	**87.2**	**12.8**	**0.0**	**92.3**	**7.7**	**0.0**
	**[8.3.2.c] maintain knowledge of research and development in eye-gaze control technology**	**70.2**	**25.5**	**4.3**	**70.0**	**30.0**	**0.0**

^a^ All 200 original items identified in Round 1

^b^ Items were retained after Rounds 2 and 3 if they were rated 7–9 (critical to include) by ≥70% of respondents and 1–3 (not critical to include) by < 15% of respondents. Shaded cells in Round 3 columns mean the item did not progress to round 3

^c^ Items in boldface text are the 94 numbered consensus statements remaining after Round 3 and which will form the basis for clinical guidelines

^d^ Items that consumers identified as priorities and which were not identified by the whole sample

### Round 2

Sixty-two people responded to 200 statements in Round 2, 49.2% response rate (Tables 2 and 3). One hundred and five statements progressed to Round 3 for further consensus building as they were rated 7–9 by ≥70% of respondents and 1–3 by < 15% of respondents. These comprised 86 statements identified when responses of all participants were considered together and a further 19 prioritised by consumers.

### Round 3

Forty-one participants responded to Round 3 of the survey, representing a response rate of 33% in relation to 126 respondents in Round 1 (Tables 2 and 3).

All participants together reached consensus on 93 statements and consumers identified one additional priority, to reach a total of 94 statements. Of the 19 statements identified as priorities by consumers in Round 2, nine were rated as priorities by all respondents in Round 3 and were amongst the final 94 statements included in the clinical guidelines. Results for all statements across all three rounds are presented in Table 4.

## Discussion

The Delphi study reported here was completed to address the gap in evidence to guide implementation of eye-gaze control technology with people with cerebral palsy and to inform the content of clinical guidelines for eye-gaze control technology implementation [6–11, 22]. Consensus was reached on 94 statements considered important to be included in these clinical guidelines. These statements were categorised in the eight main themes outlined in Table 4 and explored in detail below.

Importantly, the consensus achieved through this international, multiple stakeholder, Delphi process strongly reinforced that important outcomes for eye-gaze control technology users included engagement in play, leisure, recreation and education; enhanced quality of life and social interaction; control over the environment; and promoting communication. This is important evidence to contribute to the justification of funding for use of eye-gaze control technology as a means of accessing a broad range of technology, and potentially making a significant impact to the user and their support network.

### Themes identified in the Delphi survey to include in clinical guidelines

Several issues were common to many of the eight themes distilled from the Delphi survey. These have been extracted and presented here to reduce repetition. Optimal seating and positioning were repeatedly identified as important for supporting assessment and trial of eye-gaze control technology and to enable its ongoing use. Skilled occupational therapists and seating specialists are therefore critical members of any team supporting use of eye-gaze control technology.

The importance of clinicians possessing up-to-date knowledge of available technology and current research and development in the field was identified as important to enable matching of technology features to users’ needs. In addition, a thorough understanding of how the technology works, can be adapted to meet a person’s needs, and support their development and progress, was considered integral to successful assessment, trials and ultimately implementation and mastery. Aspects of knowledge identified as critical include how to select set up a device which incorprating calibration, configuration, good connectivity, mounting, tailoring (e.g., compensation for some visual difficulties) and how to upgrade device features.

A multidisciplinary team, with complementary skills and knowledge, working in partnership with users, families and other members of a person’s support network, was commonly identified as a priority across the themes identified in the Delphi survey and is highly consistent with recommendations in the literature [11, 40–43]. Involvement of skilled health professionals in an ongoing manner to provide education and support to the eye-gaze control technology user, and others involved in supporting them, was considered integral to a responsible assessment, trial of the technology and successful implementation in the different settings in which the technology should be used.

#### Unique features of eye-gaze control technology as an access method

Eye-gaze control technology can be expensive to purchase and complex and time consuming to implement. Its implementation needs to be tailored to each individual to best teach, support and enable a user to participate and communicate across settings and communication partners. Respondents identified features which are unique to eye-gaze control technology as a way to justify its use as an access method. These included its applicability for people with severe disability who may have no other available method of directly accessing technology, or where eye-gaze control technology increases efficiency and effectiveness of direct access to technology when compared to other indirect access methods, such as switch technology, which requires the user to scan a menu before selecting the intended target. Additional features to be considered about eye-gaze control technology are difficulty using it in outdoor lighting and the need for careful setup and placement of the device.

#### Initial assessment

One in three people with cerebral palsy has a vision difficulty [44], a consideration in using most means of AAC. Respondents identified that a vision assessment [45], including evaluation of functional vision abilities [46], acuity, visual perception and visual motor integration, was required to assist in implementing eye-gaze control technology [47]. Functional vision refers to the use of vision in everyday activities [48]. For those who are not vision specialists, aspects of functional vision can be described using the Visual Function Classification System [49] and the Eye-Pointing Classification Scale [50]. Notably, respondents clearly indicated that vision difficulties should not necessarily preclude opportunities to trial eye-gaze control technology since the technology has the capacity to be calibrated to accommodate some difficulties with vision.

Cerebral palsy frequently co-occurs with cognitive difficulties with 30–40% of children with cerebral palsy have an intellectual disability. The assessment of cognition can be challenging in people with sever motor impairment who have little or no functional speech, and to date we lack appropriate standardised tools for assessing cognition in this population [3]. Respondents did not identify that understanding of cognitive ability or ability to demonstrate cause and effect were priorities as part of an initial assessment. Respondents reported that people should not be precluded from the opportunity to trial eye-gaze control technology as, for some individuals, eye-gaze control technology may provide the means to explore cause and effect activities for the first time. However, if the purpose of introducing eye-gaze control technology as an access method is to facilitate communication, establishing a profile of cognitive and language ability is important in guiding implementation of the technology and will be a factor to consider when setting goals and managing consumer expectations. Successful AAC users and their support networks attribute success to the cognitive strengths of the user [51]. Equally, children without knowledge that their actions can cause an effect on objects, for example in the context of play, may struggle to engage meaningfully with eye-gaze control technology.

#### The trial

Several factors were considered by respondents to be important when making the decision to carry out a trial of eye-gaze control technology, including the person’s preferences, goals of using the device and the readiness of the team around the person. The literature suggests that people making informed choices about using assistive technology will need opportunities to trial different types of equipment to establish their preferences and to discuss the implications of their options [43]. The risk of device abandonment is likely to reduce when people who use assistive technologies are involved in decision making [19].

Respondents considered that trials need to be conducted in collaboration with clinicians and other stakeholders and duration of follow up needs to be long enough for the user to have the opportunity to practice and demonstrate skill development and achievement of goals. As a result, no consensus was reached on the exact length of time that is required for a trial as it will vary from individual to individual. Tailoring the length of device trials to an individual’s needs and progress is consistent with a person-centred approach to service delivery [52].

Respondents identified that goals which were collaboratively developed between the person with cerebral palsy, their support network and clinicians, and which drove a clear plan and identification of roles and responsibilities were a high priority for a successful device trial. Motivating activities for trialing and practising the technology, which were aligned with the person’s goals and interests, were reported to be crucial considerations and underscored a need for clinicians to understand the priorities and interests of the person.

#### Learning, practise and support

There was strong consensus that practise, through activities or games, was required to ensure effective implementation of eye-gaze control technology. Respondents recommended that practise should take place in regular but short sessions to optimise learning and minimise the potential impact of fatigue on performance. Of particular clinical relevance, is the recommendation that practise should be embedded in the routines of the person learning to use the technology, rather than taking place in specifically arranged practice sessions.

Ongoing education, practise and support, with an individualised plan, were recommended for the user and their support team to ensure they were competent and confident to optimise the outcomes of the eye-gaze control technology. The statements relating to these considerations are aligned with what has been reported in the general assistive technology and AAC literature [53–59]. Input from skilled professionals was considered a priority for guiding and coordinating device implementation and in tailoring and upgrading the technology for an individual. Timely, technical support and ongoing review of technology implementation, as required or requested by a user and their support network, were identified as integral to the success of eye-gaze control technology. These statements reflect those raised during a Global Research innovation and Education summit on Assistive Technology (GREAT) held in 2017 [60].

#### Outcome measures

Delphi respondents clearly indicated that the key measure of the outcome of eye-gaze control technology was the individual user’s goal achievement supplemented by observations from other relevant professionals. These goals need to be related to the purposes for which they were using the technology and co-constructed between the person using the technology and their support network. No evidence-based outcome measures were identified as a priority for use. The Investigators and Advisory Panel are concerned that funding agencies may require standardised measures with evidence of reliability and validity to supplement the individualised measures. Examples of such measures include Goal Attainment Scaling (GAS) [61], Family Impact of Assistive Technology Scale for Augmentative and Alternative Communication (FIAT-AAC) [62] and Therapy Outcome Measure (TOM-AAC) [63].

Some respondents reported that outcome measures were not accessible in their language or they were unfamiliar with the measures, indicating a need for further education and efforts to translate existing measures into languages other than English, and to develop new tools that reflect cultural and linguistic diversity.

#### Funding considerations

The way in which eye-gaze control technology is funded varies from country to country, and potentially within countries. A newly published position paper on an international framework for assuring availability and accessibility of assistive technologies supports this assertion [19]. Respondents identified that sufficient evidence needs to be provided to meet local funding criteria along with proof that a support network of health professionals and people within the user’s various environments was available and committed to supporting implementation. While attending to local funders’ criteria, respondents also strongly identified that evidence of effects across a broad range of outcomes should be offered, including participation in leisure and productivity occupations, environmental control and enhanced communication, social interaction and quality of life.

#### Preparation for possible future access

The Delphi survey sought information on actions which could be taken to develop the skills of a person and their families and caregivers, when initial trials had not been successful, but the team around the person still recognized that re-introduction of eye-gaze control technology at a later date may present new opportunities. Respondents recommended ensuring well supported seating and positioning and developing competencies to optimise the likelihood of success of a future trial. These competences included supporting the development of functional vision skills as well as enhancing communicative competence and confidence of both users and their communication partners through encouraging use of looking behaviors for communication such, as eye-pointing, in low-tech methods of communication and choice making. Different AAC systems and strategies require varying degrees of cognitive skill for successful use. Research with people with traumatic brain injury suggests that using a multimodal approach where the person is supported using low-tech solutions, such as eye-pointing to make choices, in addition to high-tech solutions [64] can help build communicative competence and confidence.

#### Limitations

While the field of assistive technology is multidisciplinary, the majority of respondents were either occupational therapists or speech and language therapists. The findings, therefore, largely reflect the particular perspectives of these health professionals who, nevertheless, are recognised as primary agents for the support of eye-gaze control technology. Fewer people with cerebral palsy completed Rounds 1 and 2 compared to other stakeholder groups, and none completed Round 3, despite the investigators’ active engagement with consumers as part of the investigator team and Advisory Panel to co-design and optimise dissemination of the surveys. The surveys were long, time-consuming and would, undoubtedly, have been taxing to complete. The impact of the limited representation of people with cerebral palsy and their communication partners on the findings of this study is unknown. Future studies will need to diligently attend to creative and accessible means for ensuring consumers’ voices contribute to findings. Although, consumers’ responses to this Delphi study were analysed separately to preference their views, communicating the findings of this study will need to be cognisant of the near-absence of consumer contributions and perspectives. Particular attention will also need to be paid to evaluating the impact of the findings for people with cerebral palsy and their support networks to ensure that knowledge translation strategies can be responsive to their needs for information and guidance about use of eye-gaze control technology.

The Delphi surveys requested information relevant to children, adolescents and adults with cerebral palsy. Although age-group-specific information was not obtained, the respondents supported eye-gaze control technology users across all age ranges and the findings offer guidance for use across the lifespan. Evaluation of our implementation of the findings will seek to identify additional considerations for different age groups and abilities.

Attrition is a feature of Delphi studies due to the multiple rounds [28]. The response rate declined with each round of this study, as expected, although the overall response rate of 33% in Round 3 is about 10 percentage points more than anticipated [65]. We sent personalised links to participants and reminder emails as a strategy to enhance the response rate. The complexity and importance of the topic meant that there were a large number of statements to rate in Rounds 2 and 3, which may have been burdensome and contributed to attrition. The proportion of participants from each stakeholder group did not change appreciably across rounds, with the exception of notably fewer educators and a reduction in numbers of people with cerebral palsy from three in Round 1 to none in Round 3. The relative contribution of the various stakeholder groups to the ratings of statements is therefore likely to be similar across rounds. The impact of attrition and the few contributions from consumers on the final selection of statements is not possible to ascertain.

## Conclusions

Participants in this Delphi process achieved consensus for 94 statements across eight domains for eye-gaze control technology implementation*: its unique role in meeting the needs of a user; initial assessment; trial; learning, practise and support; follow up; outcome measures; funding considerations and preparation for eye-gaze control technology as a possible future access method.* These statements will form the core of evidence- and consensus-informed clinical guidelines - the scope, content and implementation of which will further our knowledge in the field. These guidelines will be crafted in collaboration with an expanded Advisory Panel selected to ensure greater representation of consumers. The guidelines have the potential to evolve as our knowledge advances and evaluation of guidelines implementation informs subsequent iterations. The guidelines will be made freely available and accessible online and will contain specific guidance about implementation and evaluation of eye-gaze control technology for people with cerebral palsy including links to relevant evidence and resources.

Findings from the Delphi process strongly recommended that the clinical guidelines complement and support clinicians and consumers in their quest for finding, assessing and implementing eye-gaze control technology as an access method. The importance of realistic goals and a plan for implementation of eye-gaze control technology, along with provision of education and support for the user and their support network, including their communication partners were emphasised.

The findings may have relevance for other potential users of eye-gaze control technology such as people with Amyotrophic Lateral Sclerosis and Locked-in Syndrome. There are important differences between people with cerebral palsy who have sustained damage to the developing brain and these other, adult onsets, conditions in terms of the mechanism of physical disability, existence of co-morbidities (hearing, vision, epilepsy, cognition) and environmental exposures across the lifespan.

The findings from this Delphi study and the resultant clinical guidelines have the potential to further advance our knowledge of how to best support people with disabilities gain access to technology to enable participation in communication, work, education and leisure pursuits. Future research will focus on the free, online clinical guidelines and complementary resources developed from the findings of this Delphi study. Effective knowledge translation strategies, co-developed with people with cerebral palsy, their families, clinicians, funders and researchers, will be required to support and evaluate uptake of the clinical guidelines to ensure they reach and are implemented by intended stakeholders. The impact of implementing the clinical guidelines on outcomes considered meaningful by people with cerebral palsy and their communication partners is also required. Furthermore, future research to identify the relationships between functional vision and eye-gaze control, and between motor types of cerebral palsy, severity and eye-gaze control will assist clinicians to further tailor eye-gaze control technology implementation to optimise success.

## Supplementary Information

**Additional file 1: Supplementary file 1.** Guidance on Conducting and Reporting DElphi Studies (CREDES).

**Additional file 2: Supplementary file 2.** Open-ended questions used in the first round.

## Data Availability

All data analysed for this study are included in Table 4 in this published article.

## Abbreviations

AAC: Augmentative and alternative communication
CREDES: The Delphi process followed the principles of the guidance on conducting and reporting delphi studies
GRIPP2-SF: The Short form of guidance for reporting involvement of patients and the public 2
REDCap: Research electronic data capture
GREAT: Global research innovation and education summit on assistive technology
GAS: Goal Attainment Scale
FIAT-AAC: Family impact of assistive technology scale for augmentative and alternative communication
TOM-AAC: Therapy outcome measure augmentative and alternative communication
GMFCS E&R: Gross motor function classification system expanded and revised
MACS: Manual ability classification system
CFCS: Communication function classification system
VSS: Viking speech scale
