# Supplementary material for: Stakeholder consensus for decision making in eye-gaze control technology for children, adolescents and adults with cerebral palsy service provision: findings from a Delphi study
Source: BMC Neurol. 2021 Feb 10;21:63. doi: 10.1186/s12883-021-02077-z (PMC7874479; doi:10.1186/s12883-021-02077-z)
Supplement: Supplementary file 1 — Additional file 1: Supplementary file 1. Guidance on Conducting and Reporting DElphi Studies (CREDES). [file 12883_2021_2077_MOESM1_ESM.docx]

**Supplementary file 1**

**Guidance on Conducting and Reporting DElphi Studies (CREDES)**

| CREDES ITEMS | | EXPLANATION | Page # to be added in proof stage  Currently identified by subheading and paragraph |
| --- | --- | --- | --- |
| **Rationale for the choice of the Delphi technique** | |  |  |
| 1. | Justification. | Justification is provided for a Delphi process and particularly an online Delphi | Material and Methods section: paragraph 2:  *The Delphi process is anonymous and non-hierarchical …*  Material and Methods section: paragraph 3:  *An online Delphi process was a particularly suitable methodology …* |
| **Planning and design** | |  |  |
| 2. | Planning and process | Justification is provided for:   1. using 3 rounds 2. an undefined population rather than a pre-determined expert panel, and 3. using open-ended questions in round 1. | 1. Material and Methods section: paragraph 1:   *Three rounds are conventionally considered sufficient…*   1. Material and Methods section: paragraph 3:   *it was intended to facilitate inclusion of users of eye-gaze control technology and their communication…*   1. Survey development section: paragraph 1:   *As we had identified that there was little evidence to…* |
| 3. | Definition of consensus. | Consensus was defined as having been achieved for an item to progress to the next round when it was rated 7-9 (critically important) by ≥70% of respondents and 1-3 (low importance) by <15% of respondents. | Rating of the importance of the statements in Rounds 2 and 3 of the Rating of the importance of the statements in Rounds 2 and 3  section: paragraph 2:  *Statements rated 7-9 by ≥70% of respondents and 1-3 by <15% of respondents were considered as reaching consensus [33].* |
| **Study conduct** | |  |  |
| 4. | Informational input | The investigator team and advisory group reached consensus on the content of each round of the Delphi process and each round was fully pilot in the REDCap version accessed from the link in the email to be sent to participants. | Survey platform and pilot testing section |
| 5. | Prevention of bias | Strategies for prevention of bias:   1. One of the reasons for a consumer investigator and an Advisory Panel with diverse backgrounds was to minimise bias. 2. The surveys were completed online and anonymously so investigator bias was minimised. 3. Measures to minimise bias in the qualitative analysis of Round 1 narrative responses. | 1. Consumer and stakeholder involvement 2. Delphi process section, paragraphs 1 and 2   *A 3-round, online Delphi process was used to identify*  *The Delphi process is anonymous and non-hierarchical so it can elicit open and honest views from disparate groups*   1. Survey development section, paragraph 2:   *Thematic qualitative analysis of narrative responses to Round 1 was performed independently by three investigators* |
| 6. | Interpretation and processing of results | Our definition of consensus requiring each item to meet 2 decision rules assisted in consolidating clear consensus. | Rating of the importance of the statements in Rounds 2 and 3 of the Rating of the importance of the statements in Rounds 2 and 3 section: paragraph 2:  *Statements rated 7-9 by ≥70% of respondents and 1-3 by <15% of respondents were considered as reaching consensus [33].* |
| 7. | External validation | The final items which reached consensus will be integrated into clinical guidelines in conjunction with an expanded Advisory Panel who were uninvolved in the Delphi process. | Clinical guidelines section in Discussion |
| **Reporting** | |  |  |
| 8. | Purpose and rationale | 1. Purpose. There is limited evidence to guide practice so Delphi was used to identify expert-driven issues to include in clinical guidelines. 2. Rationale for Delphi. The Delphi process can reach a broad range of stakeholders, internationally and anonymously to identify content and to drive consensus on the most important issues to include in guidelines. | 1. Introduction section, paragraphs 3 and 4   *… there is almost no literature to guide decisions specifically about eye-gaze control technology as an access method …*  *…However, the reviews highlighted a pressing need for research be completed to inform clinical practice and support funding for this technology.*  *Evidence-based practice draws on the best available evidence, clinical expertise and the values and preferences of recipients of health care [23]. Therefore, this study aimed to build international consensus …*   1. Delphi process section, paragraphs 2 and 3   *The Delphi process is anonymous and non-hierarchical so it can elicit open and honest views from disparate groups…*  *…particularly suitable methodology for the current topic as it enabled engagement with large, diverse, international groups of participants* |
| 9. | Expert panel. | 1. In this study, participation was opened to a range of stakeholders internationally, to harness diversity of experiences, cultures, health systems and languages, and particularly the expertise of users and their communication partners.   Reporting of the following critical features of the expert panel are included   1. Inclusion criteria: *Participants were eligible to take part if they were* 2. Participant characteristics: Table 1 3. Response rates in round 1 4. Response rates in round 2 5. Response rates in round 3 | 1. Delphi process section, paragraph 3   *…intended to facilitate inclusion of users of eye-gaze control technology and their communication partners (such as parents, partners, families, caregivers and teachers), as well as clinicians, researchers, industry and funders…*   1. Participants and recruitment section   *Participants were eligible to take part if they were*   1. Table 4. 2. Results Round 1 section   *One hundred and twenty-six* participants *(86.5% female) completed Round 1 of the Delphi survey.*   1. Results Round 2 section   *Sixty-two people (86.9% female) responded to 200 statements in Round 2 (49.2% response rate)*   1. Results Round 2 section   *Forty-one participants (87.8% female)* *responded to Round 3 of the survey, representing a response rate of 33%...* |
| 10. | Description of the methods | Description of key aspects of the methods are included:   1. Synthesis of available evidence prior to study 2. Development of surveys 3. Piloting of surveys and study information 4. Number and design of survey rounds 5. Data analysis in each round   Round 1: *Thematic qualitative analysis of narrative responses to Round 1*  Rounds 2 and 3 and participant characteristics. SPSS version 25 [19] was used to calculate level of consensus and generate descriptive statistics of participant characteristics | 1. Introduction section, paragraph 3 and Discussion section, paragraph 1   *….This gap in guidance is highlighted by two recent systeatic reviews which examined: (1) the effectiveness of eye-gaze control technology, (ii) assessment and implementation strategies [17] …*   1. Survey development - Generation of Content for Round 1 section 2. Survey platform and pilot testing section 3. Delphi process section   *A 3-round, online Delphi process was used to identify,*   1. Round 1: Responses to Round 1 of the Delphi survey section   Round 2 Rating of the importance of the statements in Rounds 2 and 3 of the Delphi survey section, paragraphs 1, 2 and 3 |
| 11. | Procedure – flowchart | A flowchart (Figure 1) is included which describes the overall study process from ethics approval, survey development and piloting, interim steps of survey distribution and data processing through to future clinical guidelines development and knowledge translation. It includes dates of each stage of the Delphi process, illustrates the extent of consumer engagement and ongoing communication with study participants. |  |
| 12. | Definition and attainment of consensus. | 1. Items were identified during Round 1 for inclusion for rating for consensus building in Rounds 2 and 3. Decision rules for items to progress from Round 2 to 3 and those progressing for Round 3 to the clinical guidelines are provided 2. Additionally, items achieving consensus for consumers and which did not achieve consensus for the entire sample were progressed to subsequent round. | Rating of the importance of the statements in Rounds 2 and 3 of the Rating of the importance of the statements in Rounds 2 and 3 section: paragraph 2:   1. *Statements rated 7-9 by ≥70% of respondents and 1-3 by <15% of respondents were considered as reaching consensus [33].* 2. Rating of the importance of the statements in Rounds 2 section, paragraph 1   *Statements progressed to Round 3 for further consensus building if they were…*   1. Rating of the importance of the statements in Rounds 2 and 3 of the Delphi survey section, paragraph 2   *The process of identifying consensus statements was completed separately for two groups of participants: i) all participants together and ii) people with cerebral palsy and their families and caregivers. All statements reaching consensus from both these groups progressed to Round 3. This process ensured that the most important content from people with cerebral palsy and their families and caregivers was included for ongoing consideration.* |
| 13. | Results | All items included in each round and those retained and not retained in subsequent rounds including the summary ratings data and those identified by consumers separately are presented in Table 4 |  |
| 14. | Discussion of limitation | Limitations are discussed in a dedicated Limitations section | Limitations section |
| 15. | Adequacy of conclusions | A dedicated Conclusions section relate the Delphi findings to the proposed clinical guidelines, the impact of the guidelines on clinical practice and outcomes for consumers and provide suggestions for ongoing implementation and research. | Conclusions section |
| 16. | Publication and dissemination | This paper reports details of the Delphi study methodology and results. The publication states that the resulting clinical practice guidelines will be readily available. A knowledge translation strategy is to be developed with the expanded Advisory Group. | See Clinical Guidelines section |
