# Supplementary material for: Stakeholder consensus for decision making in eye-gaze control technology for children, adolescents and adults with cerebral palsy service provision: findings from a Delphi study
Source: BMC Neurol. 2021 Feb 10;21:63. doi: 10.1186/s12883-021-02077-z (PMC7874479; doi:10.1186/s12883-021-02077-z)
Supplement: Supplementary file 2 — Additional file 2: Supplementary file 2. Open-ended questions used in the first round. [file 12883_2021_2077_MOESM2_ESM.docx]

| Supplementary file 2. Open-ended questions used in the first round | | | |
| --- | --- | --- | --- |
| 1. Eye-gaze control technology can be used for communicating, play, learning, working and controlling the environment | | | |
|  | | 1. What is unique about eye-gaze control technology? | |
|  | | 1. What makes it different from other forms of access methods? | |
| 1. Deciding when it is time to consider eye-gaze control technology for a child or adult with cerebral palsy | | | |
|  | | 1. What factors would be considered in deciding the right time to start trialling eye-gaze control technology? | |
| 1. Assessment | | | |
| *An assessment is carried out before a child or adult with cerebral palsy starts to use eye-gaze control technology. This might include information from formal and informal assessments*. | | | |
|  | | 1. What information about the person who might use eye-gaze control technology is important to know as part of this assessment? (such as characteristics of the person and the goals they would like to achieve) | |
|  | | 1. What information about the person's environment and support network is important to know as part of this assessment? (such as home, school or work environments; other equipment; staff, family, friends and others in the community) | |
|  | | 1. What information about the technical aspects of eye-gaze control technology is important to know as part of this assessment? (such as usability, features of the device and compatibility with other technology) | |
|  | | 1. Is there other information that is required as part of this assessment? Please specify. | |
| 1. Trialling an eye-gaze control device once a person has their eye-gaze control technology | | | |
| *Before deciding that eye-gaze control technology is correct for a child or adult with cerebral palsy, there is usually a trial period to check that it is the right decision and to select the most appropriate device. During this trial period one or more eye-gaze control technology devices may be tried out.* | | | |
|  | | 1. What are the practical factors to consider in carrying out such a trial to ensure that the right decisions are made about using eye-gaze control technology? (such as time, resources and training required) | |
| 1. Deciding whether eye-gaze control technology is the right choice | | | |
|  | | 1. What are the important factors to consider in deciding whether eye-gaze control technology is the right choice for a child or adult with cerebral palsy? | |
|  | | 1. What criteria would convince a supplier, purchaser or funding body that purchasing eye-gaze control technology is the right choice for a child or adult with cerebral palsy? (This may include criteria that you believe are important, or your local or national criteria). Eye-gaze control technology may not currently be the right choice for a person but may be in the future. | |
|  | | | |
|  | | 1. What would you suggest that a person with cerebral palsy, communication partners and clinicians do to prepare for using eye-gaze control technology in the future? | |
| 1. Once a person has their eye-gaze control technology | | | |
|  | | 1. What activities and resources could be used to help a person and their communication partners learn to use the technology effectively? | |
|  | | 1. How much and what types of practice should be recommended to help a person use the technology effectively? | |
|  | | 1. How much and what types of support do you believe are required to help the person and their communication partners make effective use of the device? | |
|  | | 1. How, and how often, should a person and their communication partners review their use of use of eye-gaze control technology with a clinician? | |
| 1. Measuring the effectiveness of eye-gaze control technology | | | |
|  | | 1. How would you measure the outcomes of using eye-gaze control technology? (such as achievement of goals, or use of formal and informal assessment) | |
